# Microscale impedance measurements suggest that ionic diffusion is implicated in generating extracellular potentials

**DOI:** 10.1186/1471-2202-15-S1-P214

**Published:** 2014-07-21

**Authors:** Claude Bedard, Jean-Marie Gomes, Matthew Nelson, Pierre Pouget, Silvana Valtcheva, Laurent Venance, Yves Gioanni, Thierry Bal, Alain Destexhe

**Affiliations:** 1 UNIC, CNRS, Gif sur Yvette, France; 2 ICM, Paris, France; 3 CIRB, Collège de France, Paris, France

## 

The genesis of the Local Field Potential (LFP) highly depends on the electric properties of the extracellular medium, but such properties are still subject to a controversy because of contradictory measurements. One possibility is that the use of metal electrodes as current sources in previous studies provides non-physiological results. We tested this possibility by performing impedance measurements in conditions as close as possible to physiological conditions. We generated single-cell LFPs by injecting subthreshold inputs in single neurons using patch-clamp recordings, combined with extracellular recordings with micropipettes. Various measurement configurations show that (1) the extracellular medium has strong low-pass filtering properties and cannot be accounted by a resistive medium; (2) the frequency scaling of the filtering, as well as its phase, show that the system seems intermediate between resistive and capacitive. The extracellular impedance was also measured from in vivo experiments in rats under anesthesia. In this case, recording with intracellular (whole-cell) electrodes, together with extracellular LFP, showed results consistent with the in vitro experiments. Finally, we developed a theoretical model based on Maxwell equations, which shows that all measurements can be explained if the extracellular medium is of diffusive type (Warburg impedance). This model predicts that the phase difference between intracellular and extracellular signals should provide a signature of the physical nature of the impedance, with 45 degrees phase difference for purely diffusive type. The experiments show that indeed, the phase is that of a RC soma in series with a diffusive impedance (between 0 and -45 degrees), therefore confirming the diffusive nature of the extracellular impedance. These findings have potentially important consequences for interpreting LFP measurements and source estimation such as CSD analysis.

